# When does consciousness arise? A subcortical model of its origins

**DOI:** 10.1007/s00429-026-03113-9

**Published:** 2026-04-28

**Authors:** Saúl Sal-Sarria, Mark Solms, Oliver H. Turnbull

**Affiliations:** 1 Laboratory of Neuroscience, Department of Psychology, University of Oviedo, Oviedo, Spain; 2 Institute of Neurosciences of the Principality of Asturias (INEUROPA) and Health Research Institute of the Principality of Asturias (ISPA), Oviedo, Spain; 3 Neuroscience Institute, Department of Psychology, University of Cape Town, Cape Town, South Africa; 4 North Wales Medical School, Bangor University Wales, Bangor, UK; 5 School of Psychology, Bangor University Wales, Bangor, UK

**Keywords:** Consciousness, Affective neuroscience, Emotion, Brainstem, Periaqueductal gray, Neurodevelopment

## Abstract

The origin of consciousness constitutes one of the most enduring challenges in contemporary science, with implications for neuroscience, philosophy, medicine and ethics. Traditional accounts have emphasized the cerebral cortex as the seat of conscious awareness, largely due to its expansion and complexity in humans. However, converging evidence suggests that the foundations of consciousness lie not in higher cognition, but in more ancient subcortical systems responsible for affective experience. Central among these are the ascending arousal networks of the upper brainstem and the periaqueductal gray, which together sustain wakefulness and imbue it with valence. This reframing positions consciousness as an embodied biological function grounded in feeling, rather than as a component of higher cognition. From a developmental perspective, the relevant subcortical structures mature relatively early in gestation, raising the possibility that a primitive form of subjective feeling may emerge during the third trimester, well before cortical maturation. Such a view has far-reaching ethical consequences, informing debates on fetal pain perception, neonatal care, and the treatment of individuals with profound cognitive impairment. By synthesizing recent neuroscientific findings with developmental data, this paper argues for a subcortical basis of consciousness, and highlights the need for an integrative approach that situates the origins of consciousness within affective brainstem systems.

## Reframing the origins of consciousness

The origin of consciousness remains one of the most challenging questions in contemporary science. While various disciplines, ranging from physics to neuroscience and philosophy, have approached this issue from different standpoints (Cescon 2009; Palacios 2024), recent developments in neuroscience suggest that a meaningful account must define consciousness not as an emergent property of *cognition*, but as a more basic biological function rooted in *affect* (Damasio and Damasio 2023, 2024; Panksepp 1998; Solms 2019, 2021). In line with Nagel’s (1974) notion of *‘*what it is like’ to have experience, we define consciousness as the most fundamental form of experience, grounded in the organism’s capacity to feel. Rather than being a property of higher cognitive operations, consciousness appears to enable elementary affective states to be experienced by a subject (Solms 2019). Understanding the origins of consciousness has a number of critical and timely implications. Important issues relate, for example, to human adults with profound learning disabilities, and to patients in the late stages of dementia. Both are cases where intelligence is very limited, but consciousness and emotional experience may be substantially preserved, at least in some stages or forms of these conditions (Huntley et al. 2021). appear to be largely intact. A similar argument applies to the conscious experience of other species (Turnbull and Bär 2020; 2024). The opposite boundary condition relates to machines which are intelligent, but lack consciousness, such as our smart phones (cf. Solms 2021).

We note, importantly, that our legal and ethical systems already reflect the position that experience of affect is the critical and defining element for responsibility. It is of course illegal to mistreat or neglect human beings who are profoundly learning disabled, or patients in the late stages of dementia, or indeed many non-human animals. In contrast, it is entirely legal to mistreat or neglect a device like a smartphone or other computer, which are (in various ways) intelligent but not conscious. Indeed, it is quite legal to sell, or intentionally destroy, such a device. This is because the fulcrum of the legal and ethical question is not intelligence, but sentience, and the potential to experience distress, an issue which we have discussed at length in relation to animal minds (Turnbull and Bär 2020).

This paper reviews the important question of consciousness and affective experience in relation to another boundary case: the developing capacity for sentience. The developmental stage at which consciousness first appears has equally important ethical implications. Obvious examples would be around the abortion debate, the ethical treatment of newborn children, the question of pain relief in surgeries such as circumcision, and even for *in utero* procedures.

This question is especially pressing at the moment, because our understanding of the brain basis of consciousness and affect is now a tractable topic of scientific inquiry. Traditional models have tended to locate the capacity for consciousness in the cerebral cortex, likely –in part– due to its greater relative size (and perhaps complexity) in humans compared to almost all other species. This ‘corticocentric’ assumption is exemplified by dominant contemporary frameworks such as the Global Workspace Theory ([Bibr CR2][Bibr CR2]; Dehaene and Changeux 2011) and Integrated Information Theory (Tononi 2004; Tononi et al. 2016), both of which emphasize (fronto-parietal or posterior) cortical integration as the hallmark of conscious experience. While empirical work aligns with these perspectives regarding *perceptual* awareness (Dehaene et al. 2006; Boly et al. 2007), recent advances in network neuroscience suggest that conscious states rely less on isolated regional activation and more on dynamic inter-regional communication (Bassett and Sporns 2017; Luppi et al. 2019). Indeed, this cortical focus may stem from an anthropocentric bias (Scotto 2024). Additionally, this assumption may stem from attributing special significance to *externally*-facing sensory experiences, particularly vision, when attempting to explain our conscious awareness, given the long-established role of cortical structures in domains such as higher vision (Munk 1890).

A range of research findings suggest that the focus might be better placed on *internally*-facing sensory sources, including those that generate affect (Solms 2013, 2021).^1^ Crucially, recent theoretical advancements have cemented this perspective, arguing that the foundational source of consciousness lies in ‘homeostatic’ feelings: the interoceptive experience of the body’s state of life regulation - such as hunger, pain or thermal discomfort - (Damasio and Damasio 2023, 2024; Damasio, 2025). In this framework, consciousness is not a late-arriving cortical function, but an early-emerging process dependent on upper brainstem nuclei. Singer and Damasio (2025) recently detailed the unique physiology supporting this ‘feeling mind’. Unlike the ‘digital’, rapid, and myelinated processing of the cortex (the ‘modern mind’), the interoceptive system relies on ‘analogue’, slow, and unmyelinated signaling (including volume transmission and ephaptic coupling). This allows for a direct comingling of neural and non-neural signals in structures like the nucleus tractus solitarius (and, we propose, the periaqueductal gray), generating a continuous, sentient representation of existence which anchors the self. As a consequence, the cortico-centric view is being increasingly challenged, suggesting that the basic substrate of consciousness is subcortical, and especially linked to structures in the upper brainstem (Damasio 2010; Damasio and Carvalho 2013; Merker 2007; Panksepp 1998; Solms 2013, 2021; see also Lutkenhoff et al. 2015; 2020). As we will show below, these brainstem structures mature surprisingly early in fetal development, which of course has implications for the question of the first appearance of consciousness in the fetus.

This paper will briefly survey the current literature on the brain basis of consciousness and affect. We will then review the developmental stages at which the various components of this architecture appear *in utero*. This will allow us to draw some conclusions, and to develop evidence-based hypotheses, about the early emergence of consciousness.

## The anatomy of consciousness

Historically, a critical finding in relation to consciousness came from the identification of the ascending reticular activating system (ARAS, then described as the reticular formation) by Moruzzi and Magoun (1949). They demonstrated (among other sources of evidence) that electrical stimulation of the reticular formation in cats produced cortical desynchronization in EEG recordings, that disconnection of the cortex produced cortical synchronization, and that lesioning of the ARAS produced coma. These pivotal findings showed that cortical activation is generated *endogenously*, via these ascending brainstem pathways, rather than being dependent on externally-derived sensory input to the cortex.

Functionally, the ARAS regulates wakefulness and cortical tone through widespread projections to and from the intralaminar nuclei of the thalamus, as well as the hypothalamus and basal forebrain (Donkelaar, 2020). Importantly, these pathways rely on multiple neuromodulators, including acetylcholine, noradrenaline, serotonin, dopamine, histamine, and orexin, which modulate cortical excitability and behavioral arousal (Gazzaniga 2009; Swanson et al. 2021).

The fact that damage to the ARAS disrupts global cortical activation, resulting in coma or persistent vegetative states, has been demonstrated repeatedly (Parvizi and Damasio 2003). In contrast, even severe but selective cortical lesions do not abolish wakefulness or affective responsiveness, unless they damage the upper brainstem - by compression, for example - further emphasizing the centrality of the ARAS in consciousness (Egawa et al. 2024). The ARAS has also been implicated in emergence from anesthesia, sleep-wake regulation, and pain modulation (Bimonte et al. 2019).

Anatomically, the ARAS is a diffuse network of interconnected nuclei, embedded within the brainstem’s reticular formation, a phylogenetically ancient structure extending through the midbrain, pons and medulla (Ascenzi 2025; 1996). The diversity of nuclei, and distribution up and down the brainstem, suggests multiple evolutionary adaptations, convergent evolution, and an element of redundancy in these critical structures. The ARAS is particularly concentrated in the rostral brainstem, including the midbrain tegmentum, and it comprises more than two dozen nuclei with distinct cytoarchitectonic and neurochemical profiles (Swanson et al. 2021). Structurally, this region also contains major ascending and descending pathways, such as the spinothalamic, corticospinal and rubrospinal tracts, allowing for continuous integration of sensory-motor signals between the spinal cord and the cortex (Sengul and Watson 2015).

The ARAS itself is traditionally organized into three longitudinal columns (Caminero and Cascella 2024). The first are the raphe nuclei (median zone), serotonergic in nature, which are involved in mood regulation and nociception. The second are the gigantocellular nuclei (medial zone), essential mainly for motor coordination. Finally, the parvocellular nuclei (lateral zone) is involved in autonomic control, including respiration.

Among the key components of the ARAS is the locus coeruleus, located in the rostral pons. This nucleus is the brain’s primary source of noradrenaline, and it plays a crucial role in regulating cortical tone, arousal, vigilance and stress (Maness et al. 2022). Structures such as the pedunculopontine tegmental nucleus (PPT) and the laterodorsal tegmental nucleus (LDT) are, in contrast, cholinergic. Together with other brainstem nuclei, the locus coeruleus contributes to a neuromodulatory system that broadcasts signals to widespread cortical and subcortical regions, to maintain a globally responsive brain state (Scammell et al. 2017).

This anatomically distributed system appears to enable the integration of both internal and external stimuli necessary for sustaining a state of wakefulness, responsiveness and arousal (Damasio, 2010). Despite its anatomical diversity, it appears to develop into a functionally unified system, which becomes the foundational upper brainstem platform for the emergence of consciousness (Damasio 2010; Damasio and Damasio 2023, 2024; Damasio 2025).

However, arousal represents only one dimension of the conscious state. A related component of consciousness is that it is not *neutral*; it carries valence: a sense of pleasure or unpleasure, which permeates experience (Damasio and Damasio 2023; 2024; Solms 2021; Turnbull and Bär 2020).

### The anatomy of affect

While the brain basis of consciousness was largely understood by the middle of the last century (Moruzzi and Magoun 1949), the brain basis of affect has only recently become a tractable topic for neuroscience (Damasio and Damasio 2023, 2024; Panksepp 1998). Research has focused on a range of subcortical brain structures, such as the amygdala, hypothalamus and nucleus accumbens (Damasio and Damasio 2023, 2024; Panksepp 1998). However, the core structure for affect appears, again, to lie in the upper brainstem, adjacent to the cerebral aqueduct which connects the 3rd and 4th ventricles. This gray structure, located directly around the aqueduct, provides the basis for its name: the periaqueductal gray (PAG).

Anatomically, the PAG has a cylindrical formation. It is topographically divided into longitudinal columns, dorsomedial, dorsolateral, lateral, and ventrolateral, based on their position relative to the aqueduct, and their distinct cytoarchitectonic and neurochemical properties (Beitz 1985; Stempel 2024).

Importantly, the PAG is densely interconnected with both the ascending and descending reticular activating systems (Lim et al. 2009). It receives afferent projections from numerous other brain regions, notably the hypothalamus, amygdala, prefrontal cortex and insular cortex, and it projects to key medullary centers involved in cardiovascular and nociceptive control (Carrive and Morgan 2004; Hemington and Coulombe 2015). Functionally, it constitutes a major hub in the pain matrix: it integrates nociceptive signals from the spinal cord, relays them to thalamic nuclei, and exerts descending analgesic control through projections to the nucleus raphe magnus, modulating dorsal horn activity. This influence is mediated by a diverse set of neuromodulaters, including opioids, glutamate, neurotensin, GABA and monoamines. A tonic GABAergic interneuron network within the PAG provides inhibitory control, which is lifted (e.g., via opioid action) to enable analgesia (Tobaldini et al. 2019).

Beyond nociception, the PAG plays a central integrative role in so-called ‘survival-related’ behaviors, including autonomic regulation, vocalization, sexual receptivity, and the processing of fear and anxiety (Benarroch 2012, 2020). Its columnar organization reflects a functional gradient: dorsolateral stimulation typically triggers ‘active’ defensive responses (flight, vocalization), whereas ventrolateral activation induces ‘passive’ states such as freezing and immobility (Tsang 2018). These responses are mediated by interacting neuromodulatory systems (serotonergic, GABAergic, and CCKergic) distributed across subregions (Behbehani 1995).

Damage to specific subregions of the PAG disrupts pain perception, autonomic homeostasis and instinctive behaviors (e.g., maternal care, aggression), leading to pathologies such as chronic pain syndromes, panic disorder and emotional blunting (Jang et al. 2016; Zhang et al. 2024). Moreover, as highlighted by Panksepp (2016), extensive bilateral lesions of the PAG have been shown to abolish nearly all behavioral indicators of consciousness, in several mammalian species (Bailey and Davis 1942, 1944), as well as in rare documented cases in humans (Schiff 2007, pp. 599–600). Lesions localized to the PAG abolish emotional expressivity without affecting wakefulness, leading to what Motta et al. (2017) describe as an “affective blank stare,” more commonly known as ‘non-responsive wakefulness’.

## Dual systems

Taken together, the ARAS and PAG represent a complementary, or dual, system at the core of the anatomy of consciousness. The ARAS is prerequisite for cortical and subcortical activation, integrating arousal and affective tone to maintain the brain in a responsive, globally integrated state (Damasio and Damasio 2023, 2024). However, arousal alone does not amount to experience. The PAG acts as a foundational substrate for valenced affective consciousness. By integrating interoceptive signals, visceral arousal, and environmental threats, it generates the raw affective qualia, the biological values, that imbue wakefulness with personal significance (Damasio and Damasio 2023, 2024). It is less clear whether specific sites in the PAG generate specific basic emotions, such as fear or anger, but its overarching role in all valenced affectivity seems clear (Panksepp 2016). In sum, the ARAS maintains the living system’s alertness, while the PAG ensures that this alertness is imbued with *feeling*. Together, they constitute the minimal neural architecture for sentient awareness: anchoring consciousness not in cognitive representations, but in embodied affectivity.

On the basis of these neuroscientific data, it becomes possible to investigate when and how these brainstem structures emerge during human maturation. The following section traces the anatomical, functional and connectivity-based maturation of the ARAS and PAG, from the earliest stages of embryogenesis through fetal development and, when necessary, into the neonatal period. At each maturational stage, we will also seek to link brain development with any behaviors which potentially offer an opportunity to understand function.

## The development of consciousness

Critically, the human nervous system develops along a caudal-to-rostral trajectory: progressing from the spinal cord and brainstem toward more rostral and phylogenetically recent brain areas, such as the cortex (Stiles and Jernigan 2010; Thiebaut de Schotten et al. 2017). Brainstem regions, like the ARAS and the PAG, therefore emerge ontogenetically much earlier than forebrain structures, such as the basal ganglia and cerebral cortex (Badre and D’Esposito 2009).

As a result, the structural and functional organization of the brainstem precedes that of higher cortical regions by a significant margin. As we will show below, the brainstem has its basic structure in place by the middle weeks of gestation (Zhang et al. 2011), and its structural development is in most respects *complete* by normal term birth (Dubois et al. 2014; Deoni et al. 2015; Joseph 2000; Zhang et al. 2011).

By comparison, the cerebral hemispheres are much slower to develop (Dubois et al. 2014). At birth they are only a fraction of their later size, and lack much of their distinctive pattern of gyri and sulci. Critically, cortical myelination, even for primary motor and sensory areas, does not show substantial development until *after* birth (Deoni et al. 2015; Dubois et al. 2014; Eyre et al. 2000). That is, the cerebral cortex follows a protracted course of development, with synaptogenesis, pruning, and myelination extending into adolescence and early adulthood. Recently data suggests that full cortical maturation, particularly in prefrontal regions responsible for executive control, may not be achieved until the third decade of life (Mills et al. 2016; Tamnes et al. 2017; Steinberg 2007). However, as Singer and Damasio (2025) note, the subcortical ‘feeling mind’ does not require this high-speed insulation. Instead, it relies principally on unmyelinated or lightly myelinated fibers (such as those in the vagus nerve and the ascending reticular pathways) to support continuous, analogue signaling. This distinction is crucial: the lack of cortical myelination in the fetus, often cited as evidence *against* fetal consciousness, is in many respects not relevant to the functionality of these primitive, unmyelinated subcortical systems.

**Fig. 1:**
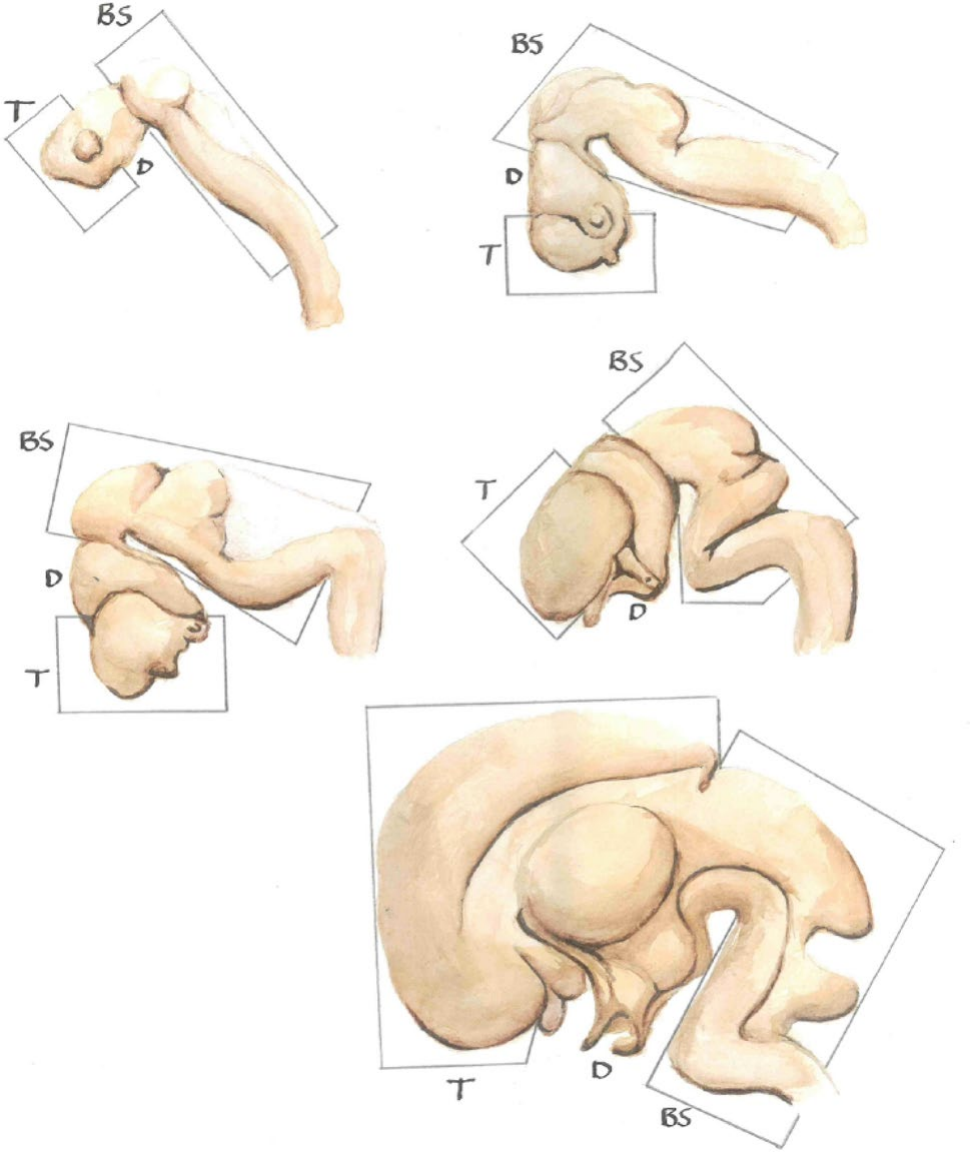
Caudal-rostral development of the human brain *in utero*, with the brainstem (labelled BS) dominating in early maturation, and the telencephalon (labelled T, which will become the cerebral hemispheres) growing substantially in the later stages. The area between the two is the diencephalon (labelled D, which will become the thalamus and hypothalamus)

Thus, brainstem structures like the ARAS or the PAG not only emerge earlier in development, but also serve as the primary functional substrate, supporting and shaping later cortical development. Overall, we have a hierarchical organization in which the initially developing system, the brainstem, organizes the function and structure of later developing systems such as the cortex (Solms 2021; Turnbull and Salas 2021).

However, cognitive neuroscience has had a long-standing focus upon these newer brain regions, with less attention being focused on the subcortical structures, and especially a neglect of the brainstem (see Bear et al. 2020; Gazzaniga 2009; Lagercrantz 2016). Thus, we have a range of structures involved in affective and cognitive processes “found from the lower brainstem up to the telencephalon, [which] are probably not even on the radar of most cognitive neuroscientists” Janacsek et al. (2022, p.365).

In the following section, we attempt to outline the developmental trajectories of the nervous system from conception, with particular emphasis on the brainstem.

In early embryogenesis (3–4 weeks of gestation) the neural tube forms; the primordial structure from which the brain’s major divisions will develop: the prosencephalon, the mesencephalon (which will later contain the PAG), and the rhombencephalon. The rhombencephalon will subsequently differentiate into the metencephalon (which gives rise to the pons and cerebellum) and the myelencephalon (which forms the medulla oblongata), both of which are essential components of the brainstem. The ARAS will be distributed across these latter regions. At this early stage, the brainstem is among the first neural structures to emerge, and it begins to function as a developmental scaffold, guiding the hierarchical maturation of higher-order structures such as the forebrain and cerebellum (Stiles and Jernigan 2010). At this stage, there is no behavioral evidence to suggest any form of mental activity, and this is consistent with the anatomical data.

By weeks 6–8 of gestation, differentiation of the brainstem regions begins. The first neurons of key nuclei such as the *raphe nuclei* (serotonergic) and the *locus coeruleus* (noradrenergic) emerge, laying the neurochemical foundations of the ARAS. This constellation of brainstem nuclei and diffuse neurons is embedded within the tegmental and medullary regions of the pons and midbrain (Edlow et al. 2012). These neurons are among the first in the brain to differentiate. For instance, serotonergic raphe neurons in the medulla emerge as early as 7 gestational weeks (GW) and form identifiable clusters by 10–12 GW (Kinney et al. 2007). Due to the ARAS’s diffuse and heterogeneous organization, pinpointing a single starting point for its development is not feasible. However, this early window provides a solid foundation for further investigation into its ontogeny and functional integration. Again, up to this point in gestation there is no compelling behavioral evidence of mental activity, despite the ongoing structural maturation.

Monoaminergic neurotransmitters produced in the brainstem act as early neurotrophic factors, supporting cortical neuronal migration and lamination (Dadalko andTravers 2018). This illustrates the ontogenetic dependence of the cerebral cortex on brainstem structures - an asymmetrical, but bidirectional, relationship that may also be reflected at the psychological level. Just as cortical circuits require subcortical input to organize structurally, higher cognitive functions depend on more primitive brainstem systems to be functionally and affectively grounded.

On the other hand, although precise information on this structure is more limited, Verney et al. (1991) report the presence of tyrosine hydroxylase-immunoreactive (TH-IR) neurons in the mesencephalic tegmentum and roof plate as early as the 6th GW; regions that anatomically correspond to the area surrounding the cerebral aqueduct and encompass the developing PAG. Tyrosine hydroxylase is a rate-limiting enzyme in the biosynthesis of catecholamines, including dopamine and noradrenaline, and its early expression suggests that the PAG begins to acquire neurochemical functionality at a stage when cortical structures remain largely immature.

By weeks 8–12 of gestation, projections from the *locus coeruleus* begin to extend diffusely toward higher structures such as the cortex, thalamus, cerebellum and spinal cord. In parallel, axons from the *raphe nuclei* reach supratentorial levels, forming an initial functional outline of the ARAS. Noradrenergic neurons of the *locus coeruleus*, located in the rostral pons, are already present at this stage: fluorescence histochemistry has identified mature catecholaminergic fibers in human fetuses by 12 GW (Choi et al. 1975). By approximately 17–18 GW, these neurons express high levels of tyrosine hydroxylase, indicating robust noradrenergic differentiation. Alterations in *locus coeruleus* development during this window have been associated with sudden perinatal death (Lavezzi et al. 2005).

These ascending pathways help establish early thalamocortical connectivity. Even before synapses are fully functional, spontaneous activity in the serotonergic and noradrenergic brainstem shapes developing sensory circuits, including auditory and visual systems (Clause et al. 2014; Hanswijk et al. 2020; Rockhill et al. 2009). This suggests that the ARAS begins to exert influence on the developing forebrain as early as the late first trimester, well before cortical layers are fully organized. Again, at this stage, there is no behavioral evidence to suggest any form of mental activity (before week 12).

By weeks 12–15 of gestation, an increase in spontaneous electrical activity is detected in the brainstem, reflecting the onset of large-scale neural network organization and early behavioral expression (Momose-Sato & Sato, 2013). The serotonergic and noradrenergic networks begin to show functional properties, participating in the modulation of primitive states such as sleep, wakefulness, and basic attentional levels.

Supporting this, within the midbrain and pons, other ARAS components such as the pedunculopontine nucleus and the laterodorsal tegmental nucleus - cholinergic centers crucial for arousal and REM sleep - also differentiate (Polli and Kohlmeier 2022). Neuronal populations within the brainstem begin to cluster into identifiable nuclei: serotonergic somas organize into distinct raphe nuclei (Hanswijk et al. 2020), and the locus coeruleus becomes visible as a pigmented complex by mid-gestation (Lavezzi et al. 2013). This intrinsic activity not only influences subcortical targets but also contributes to cortical organization through serotonergic signaling that supports axonal targeting and synaptogenesis (Trakhtenberg and Goldberg 2012).

In sum, at this stage, there is at least some neurophysiological evidence which might be taken to suggest a primitive form of mental activity. However, this evidence is not powerful, and further research would be needed to see if there is more solid justification for claims about the presence of consciousness as early as 15 gestational weeks.

From week 20 to week 28, the basic anatomical and functional organization of the ARAS becomes consolidated. Modulatory transmission to the thalamus and cortex is established, enabling the emergence of a rudimentary wakeful state. Converging behavioral and physiological findings indicate that, from around week 23, rapid eye movement (REM) begins to consolidate, with REM-like activity emerging during intrauterine sleep (Inoue et al. 1986; Nakahara et al. 2022). In humans, EEG recordings from 24 weeks reveal spontaneous activity transients; bursts of high-amplitude activity linked to the maturation of thalamocortical and cortico-cortical connections, often triggered by subplate activity and associated with fetal twitches (Vanhatalo and Kaila 2006). By 28 weeks, cyclic alternations between active and quiet sleep states are identifiable, marked by coordinated patterns of eye movements, body movements, and irregular breathing and heart rate (Horne 2018, p. 450).

In addition, the fetus in this period displays tactile responsiveness, with increased arm, head and mouth movements in response to maternal abdominal touch (Marx and Nagy 2015). Heartrate variability during fetal respiratory movements reflects reliable vagal activity and more mature autonomic regulation (Rahbek-Zizzo et al., 2022), and its developmental trajectory between 20 and 28 weeks predicts later cognitive and language outcomes (DiPietro et al. 2007). By 23–28 weeks, fetuses can also exhibit habituation to repeated vibrotactile stimuli, progressively reducing and eventually ceasing their motor response, followed by dishabituation to novel stimuli, indicating early sensory filtering and basic learning processes (Leader et al. 1982).

This is also the period when the brainstem begins to myelinate at scale, ahead of all other brain regions. Cortical areas connected to it, such as the primary auditory cortex, show early maturation due to this myelination. The progression closely aligns with key developmental milestones (Dietrich et al. 1988). Notably, myelination begins early in the motor and sensory roots of the cord, special sensory pathways, and the brainstem; regions essential for reflex activity and fundamental survival functions (Kinney et al. 1988; Yu et al. 2023).

At the same time, neurochemical maturation of adjacent midbrain structures is underway. The PAG, for instance, shows expression of multiple receptor types - including nicotinic, muscarinic, serotonergic, opioid, and kainate receptors - by 19 gestational weeks. Although their developmental trajectories vary, this diversity indicates that the PAG is already engaged in functional signaling during the second trimester (Reddy et al. 1996). In sum, there is convincing evidence for the neural substrate of consciousness, and its behavioral correlates, in weeks 23–28. For a *strong* claim of the existence of robust sentient experience, we again consider that this phase lacks a sufficiently powerful evidence base, though we would not rule out more convincing evidence being presented in the future.

During the third trimester (28–40 weeks), the ARAS and the PAG continue their maturation, now at a much larger scale than weeks 23–28, taking the key components of ARAS and PAG to levels much more consistent with completed development. This period is marked by an acceleration in synaptogenesis, myelination, and the refinement of functional connectivity within the brainstem. As the density and complexity of ascending projections increase, the physiological substrates for arousal and affective regulation become more robust.

At this stage, there is clear neurophysiological evidence which has parallels in the post-partum period, and in the later developing child. EEG activity now exhibits clearly organized sleep–wake cycles, including well-differentiated alternating REM and non-REM phases, a developmental progression from the immature REM-like activity observed from 23 weeks onward. These cycles are modulated in part by the ARAS and pontine reticular nuclei. Notably, from 27 to 30 weeks onward, a marked increase is observed in the correlation between respiratory movements and REM phases, suggesting improved integration between brainstem respiratory centers, reticular arousal systems, and the motor expressions of sleep (MacLean et al. 2015; Okai et al. 1992).

In parallel, serotonergic transmission, originating largely in the raphe nuclei, contributes to oligodendrocyte function and promotes widespread myelination (Fan et al. 2015), enhancing the efficiency of signal propagation across ascending pathways. This neuromodulatory support is not only critical for arousal and attention but may also influence the structural integration of sensorimotor and affective circuits.

In addition, recent studies in humans suggest that, during this period, functional associations between fetal autonomic activity and brain connectivity can already be detected. In particular, fetal mean heartrate and heartrate variability have been found to correlate with functional connectivity between the medulla, hypothalamus and dorsal anterior cingulate cortex; key regions for autonomic and emotional self-regulation (Pollatou et al. 2024). These findings indicate that the functional organization of the ARAS, the PAG and related structures not only advances anatomically, but also begin to integrate interoceptive information well before birth.

In brief, then, there is a growing body of behavioral and neurophysiological evidence in the third trimester to suggest mental activity. Not only are these based on a move towards completed structural developments, but also in behavioral terms. These take the form of sleep-waking cycles, an interest in novel events, and withdrawal from aversive stimuli. All are hallmarks of consciousness in adults, associated with affective experience. Spontaneous motor behaviors, sleep-wake regulation, and autonomic coordination (previously shaped by subcortical circuits) have now become integrated with an increasingly responsive cortical system. Thus, the ARAS, through its ascending projections to the thalamus and cortex, facilitates the transition from internally generated rhythmic activity to stimulus-based responsiveness, enabling basic states of alertness and environmental engagement (Lin et al. 2011; Maldonato et al. 2011).

Although less directly observable, the PAG continues to refine its integrative role in autonomic regulation, affective reactivity, and behavioral modulation. Its connectivity with limbic and cortical structures becomes more functionally significant, suggesting a growing capacity for the modulation of affective states (Weis et al. 2021).

The connectivity patterns observed in neonates suggest that individual differences in the functional organization of the brainstem and ARAS associated regions may be partly shaped by fetal autonomic activity during gestation. This early link between physiological signals and brain connectivity reinforces the idea that the ARAS functions as a foundational platform for emotional and behavioral regulation from birth, consolidating its role as a central structure in the transition toward more complex forms of conscious experience (Pollatou et al. 2024).

## The third trimester or birth?

Together, these developments lay the neurobiological groundwork for the progressive emergence of consciousness as a unified, embodied and affect-laden state. Rather than appearing abruptly, consciousness unfolds along a continuum, rooted in brainstem systems that are already active before birth. However, if we are required to identify a specific time period as the origin of these mental experiences, the third trimester of pregnancy would be the most likely candidate.

This framing invites a reconsideration of Lagercrantz’s (2016, 2025) account of post-natal awakening. He emphasizes that the moment of birth is marked by a noradrenergic surge from the locus coeruleus, leading to a dramatic change in arousal. The newborn becomes alert, cries within seconds, and responds vividly to external stimuli. According to this account, the locus coeruleus seems to “wake up” the brain at birth, and suggests that onset of consciousness requires, as a necessary condition, the activation of this brainstem nucleus.

This view may give the impression that consciousness begins at birth, triggered by a single neurochemical event. Yet the available evidence suggests otherwise. Responsivity, both behavioral and neural, is already present well before delivery (Leisman et al. 2024), and there is the emergence of structured EEG patterns around 24 weeks. This suggests the rudiments of consciousness may begin to emerge toward the end of the second trimester of gestation, and that they are clearly present in the third trimester. There is no doubt that birth itself generates substantial neurochemical changes. However, the available evidence suggests strongly that mental life precedes normal term delivery.

## Powerful dissociations

The early development of the brainstem, with the much later development of the cerebral hemispheres, has important implications for mental development. In particular, it produces some strong dissociations in function, even in a single psychological domain. These dissociations are especially notable in cognition, which relies more heavily on the (later developing) cortex. We briefly review three examples of such dissociations.

Our first example relates to affect. As discussed above, children are, from birth, easily overwhelmed by powerful feelings. Indeed, a substantial part of parenting is the outsourcing of the management of these feelings, given that the child lacks ability to contain them. As discussed above, the early arrival of affective experience is a consequence of the upper brainstem origins of powerful feeling states. However, we also know also that children progressively become more able to manage and constrain these powerful feelings with time, in the evolving process of affect regulation. The process takes many years to develop (Cole et al. 2011), and (as discussed above) arguably proceeds even into the third decade of life. The brain basis of affect regulation is increasingly well understood, and appears at its highest level to be a (largely, but not entirely) cortical phenomenon, linked especially to the frontal lobes (bilaterally, depending on affect regulation strategy) and the insula (Salas et al. 2013, 2016; Turnbull and Salas 2021). Thus, the delay in development of these cortical brain regions (Palser et al. 2025) seems the most plausible explanation for the gradual development of affect regulation across childhood, in contrast to the early appearance of raw affective experience.

Our second example relates to the origins of the self – the sense of *being* which places us at the heart of an experiential world. The position of affective neuroscience on this seems clear: the origin of the self coincides with the origin of the consciousness, mediated by a group of upper brainstem systems. Panksepp, for example (1998, pp.311–313), describes an upper brainstem ‘SELF’ system (using his characteristic upper-case terminology). Similarly, Solms, following Merker (2007), describes the brain’s ‘decision triangle’, which again lies in the upper brainstem, as the core of the ‘self’ (Solms 2021, p.139). Damasio (2010, p.84) makes a similar claim, especially in relation to the superior colliculus and related structures.

However, the developmental psychology literature traditionally uses a different touchstone of the self: the mirror self-recognition measure, through the ‘red spot’ or ‘rouge’ test devised by Gallup (1970). Here, the parent unobtrusively marks the child with a red spot, the child looks in a mirror, and the outcome measure is whether the child explores the new spot. Developmentally, younger children regard the figure in the mirror as a ‘sociable playmate’, and awareness of the spot on themselves only appears by circa 18–24 months (Priel and de Schonen 1986). The same developmental achievement is seen in some adult mammal and bird species (see Turnbull and Bär 2020 for a review).

Thus, we have a difference of opinion between two literatures. The affective neuroscience community make the case for selfhood from birth, while the developmental psychology community, using mirror-self recognition, suggest that this skill takes several years to acquire. The gap between the two is probably attributable to the fact that mirror self-recognition is a complex visuo-spatial task. For the young child, on our view, the self is located, with unfailing consistency, inside the body. Suddenly, with exposure to a mirror, the self continues to exist, but is now simultaneously located elsewhere in space - which is a complex concept to grasp. These spatial skills (technically, allocentric coding) are in large part cortically mediated (notably by parietal and related structures), and of course develop slowly. Thus, the temporal gap between developing these two very different senses of ‘self’ reflects the capabilities of upper brainstem versus cortical systems, on quite different developmental trajectories.

Finally, some interesting effects in the domain of memory. The ability of young children to consciously remember material, described as recent episodic memory, is famously modest. It is effectively at a floor of performance until the age of three or four, and even then, the child’s episodic memory heavily under-performs relative to the abilities of adults (Benear et al. 2025). This is the basis for the well-known phenomenon of infantile amnesia. The most plausible candidate explanation derives from the late development (across the first few years) of the hippocampus and related structures (Nadel and Zola-Morgan, 1984; Payne et al. 2010). Notably, however, young children are strikingly able to deploy *other* types of memory, for example acquired non-declarative emotional responses, such as those used to form attachment relationships (Bowlby 1988). Indeed, non-declarative memories can form, even *in utero*, on the basis of pleasurable or unpleasurable associations to tastes or sounds (see e.g. Poćwierz-Marciniak & Harciarek, 2021). In brief, we find a powerful dissociation between different categories of memory in childhood, which is likely to reflect the relative maturation of affective versus cognitive brain regions.

### Ethical and clinical implications

The subcortical model of consciousness proposed here has direct implications for clinical ethics, particularly regarding the debate on fetal pain and neonatal analgesia, though a detailed discussion is beyond the scope of this paper. If the neural substrates of sentience are functional by the third trimester, as the anatomical and physiological evidence suggests, then the capacity for suffering does not await cortical maturation. This challenges the prevailing view that cortical ‘readout’ is required for pain to be experienced as a feeling state. Consequently, the management of analgesia in fetuses and neonates during invasive procedures becomes an ethical imperative, driven by the likelihood of genuine felt experience, rather than merely suppressing nociceptive ‘reflexes’.

This framework also offers a clearer lens for understanding disorders of consciousness. For example, it aligns with clinical observations that children with hydranencephaly display not only wakefulness but also appropriate affective responses, reinforcing the view that the machinery of ‘feeling consciousness’ resides in the brainstem (Merker 2007; Shewmon et al. 1999). Indeed, Shewmon documented that such children, despite the absence of cortical hemispheres, demonstrate clear signs of sentient behavior, including musical preferences, responses to pain, and discrimination regarding familiar people. Recognizing the subcortical basis of these states may lead to more accurate diagnoses and compassionate care for patients who retain affective consciousness despite severe cortical impairment – though, again, this is not the place for a detailed discussion of the issue.

## Conclusion

Taken together, the evidence reviewed here suggests that consciousness does not arise with the maturation of the cerebral cortex, but rather with the maturation and functional integration of subcortical systems, most notably the ascending reticular activating system and the periaqueductal gray. These structures, located in the upper brainstem, mature much earlier than forebrain regions such as the cortex, and appear to be sufficient, both phylogenetically and ontogenetically, to support elementary forms of sentient being.

Anatomically diffuse, yet functionally coherent, the ARAS sustains a globally alert state, by broadcasting neuromodulatory signals throughout the brain. The PAG, in turn, imbues this arousal with affective tone, by integrating interoceptive inputs and generating raw valenced qualities, which give wakefulness its subjective values. Through this dual system, the ARAS ensures that the brain is *awake*, and the PAG ensures that it is awake for someone who *feels*. Consciousness, therefore, is not merely a representation of the world, but the powerfully felt experience of *being* in it, for better or worse.

From a developmental perspective, these systems differentiate fairly early during embryogenesis, and appear to begin functioning well before birth. While full-fledged consciousness likely develops along a continuum, the presence of a degree of completed anatomical development, as well as sleep-wake cycling, nociceptive processing, and autonomic-affective coupling in the third trimester, suggests that a primitive but genuine form of subjective experience is already present during late gestation.

As stated above, this topic remains under-investigated. Future research would do well to adopt a more integrative, subcortical-focused approach to neurodevelopment. This includes applying fetal neuroimaging, transcriptomic profiling, and advanced histological techniques to map the maturation of the ARAS and PAG in early gestation. Special attention should be given to their connectivity and functional coupling, which remain largely unexplored.

This paper offers a perspective on the importance of early-maturing brain regions for the origins of consciousness. We have a wave of new data, and new conceptual frameworks, in developmental neuroscience. These are shaping consciousness research, with important implications for bioethics. If sentience does not depend upon the cortex, but on the early coupling of arousal and affect, then the question “When is the mind born?” may find its answer, not in cerebral cognition, but in embodied affectivity. The data suggest that consciousness emerges much earlier in development than is usually assumed.

## Data Availability

No datasets were generated or analysed during the current study.

## Footnotes

1 However, not all affect is interoceptive. Consider the examples of pain and startle in response to external stimuli.
